# Association of lncRNA *CCAT2* and *CASC8* Gene Polymorphisms with Hepatocellular Carcinoma

**DOI:** 10.3390/ijerph16162833

**Published:** 2019-08-08

**Authors:** Edie-Rosmin Wu, Ming-Ju Hsieh, Whei-Ling Chiang, Kuan-Chun Hsueh, Shun-Fa Yang, Shih-Chi Su

**Affiliations:** 1 Institute of Medicine, Chung Shan Medical University, Taichung 402, Taiwan; 2 Division of General Surgery, Department of Surgery, Lin Shin Hospital, Taichung 408, Taiwan; 3 Cancer Research Center, Changhua Christian Hospital, Changhua 500, Taiwan; 4 Graduate Institute of Biomedical Sciences, China Medical University, Taichung 404, Taiwan; 5 School of Medical Laboratory and Biotechnology, Chung Shan Medical University, Taichung 402, Taiwan; 6 Division of General Surgery, Department of Surgery, Tungs’ Taichung MetroHarbor Hospital, Taichung 433, Taiwan; 7 Department of Medical Research, Chung Shan Medical University Hospital, Taichung 402, Taiwan; 8 Whole-Genome Research Core Laboratory of Human Diseases, Chang Gung Memorial Hospital, Keelung 204, Taiwan; 9 Department of Dermatology, Drug Hypersensitivity Clinical and Research Center, Chang Gung Memorial Hospital, Taipei, Linkou and Keelung 204, Taiwan

**Keywords:** long non-coding RNA, *CCAT2*, *CASC8*, polymorphism, hepatocellular carcinoma

## Abstract

The worldwide incidence of hepatocellular carcinoma (HCC), the major histological type of primary liver cancer, is heterogeneous due to the variable prevalence of etiological factors, indicating a correlation of HCC risk with genetic variations among individuals. Among long non-coding RNAs (lncRNAs) located in the chromosome 8q24 loci and involved in the carcinogenesis are colon cancer associated transcript 2 (*CCAT2*) and cancer susceptibility candidate 8 (*CASC8*). In this study, the association of *CCAT2* and *CASC8* gene polymorphisms with the occurrence of HCC was explored between 397 HCC patients and 1195 controls. We found that carriers of rs6983267 GG in *CCAT2* were more susceptible to HCC, with the odds ratio (OR) and adjusted odds ratio (AOR) being 1.532 (95% CI, 1.103–2.129; *p* = 0.011) and 1.627 (95% CI, 1.120–2.265; *p* = 0.033), respectively. Moreover, for patients stratified by age (under 65), gender (male only), or status of drinking (habitual drinkers), a protective effect of *CASC8* rs3843549 on presenting high Child–Pugh scores, metastatic vascular invasion, or large-size tumors was observed in a dominant model. Collectively, our data reveal association of *CCAT2* and *CASC8* gene polymorphisms with the occurrence and progression of HCC.

## 1. Introduction

Hepatocellular carcinoma (HCC), the major histological type of primary liver cancer, is the sixth most frequent type of malignancies with not only a steep death rate [1] but also a highly heterogeneous global prevalence [2]. Although most HCC develops in subjects with a background of established hepatic conditions [3], numerous risk factors, such as exposure to iron overload, prolonged infection with hepatitis B or C virus (HBV or HCV), use of excess tobacco and alcohol, and aflatoxin B [4,5], are known to contribute to the complex process of liver tumorigenesis. Notably, findings of numerous studies have indicated that single-nucleotide polymorphisms (SNPs) alone or jointly with other established risks mediate the carcinogenesis of HCC in an ethnicity-specific manner [6,7,8]. These data implicate genetic polymorphisms that affect inflammation, DNA repair, oxidative stress, iron metabolism, immune modulation, and intracellular signaling as predisposing factors for hepatic neoplasms and in part account for the variation in disease incidence worldwide.

Recent investigations of functional genomics have demonstrated that a substantial portion of human genomic DNA would be transcribed to RNA but merely 1.5% is encoding for proteins [9,10]. Thus, a paradigm shift into current knowledge regarding the functional role of the non-coding transcriptome emerges, in large with the attention to a growing class of long non-coding RNAs (lncRNAs). The number of lncRNAs is estimated to outrun the number of the coding genes [9], and their functionality is known to be versatile based on their capability of controlling transcription, translation, and protein function at multiple levels [11]. In addition, an increasing number of lncRNAs has been casually relevant to a myriad of pathological conditions [12], including malignancies. Findings from current studies on cancer transcriptome and genome have correlated the intricate profile of lncRNA expression with malignant transformation and unveiled an expanding catalog of functional alleles located in the lncRNA genes [13,14], indicating a role of dysregulated lncRNAs in tumorigenesis.

Colon cancer associated transcript 2 (*CCAT2*), a newly discovered lncRNA located at a recurrently amplified region (8q24) in cancers, was demonstrated to promote tumor growth, metastasis, and chromosomal instability in colon cancer via upregulation of the proto-oncogene, *MYC* [15]. In addition to being oncogenic in colon cancer, upregulation of *CCAT2* has been observed in cell lines and clinical specimens of diverse malignancies [16]. Moreover, in different populations, *CCAT2* gene polymorphisms have been linked to the risk or therapeutic response for numerous cancer types, including colon [17,18], kidney [18], thyroid [18], larynx [18], lung [19], and myeloid [20] cancer in different populations.

Cancer susceptibility candidate 8 (*CASC8*) is another lncRNA identified within the 8q24 gene desert. Through attenuating the glycolysis, *CASC8* was reported to suppress the proliferation of bladder cancer cells [21]. Similar with *CCAT2*, robust association of *CASC8* gene variations was observed with colon cancer [22,23,24] and other tumor types [25,26,27]. Nevertheless, the correlation of *CCAT2* and *CASC8* gene polymorphisms with HCC risk remains unexplored. Here, we performed a hypothesis-driven case-control investigation to explore the impact of *CCAT2* and *CASC8* SNPs on the susceptibility to hepatic tumors and have detected their associations with the occurrence and clinical indices of HCC.

## 2. Materials and Methods

### 2.1. Study Cohort

Three-hundred ninety-seven cases with HCC, together with 1195 controls, were enrolled from 2006 to 2018. This study was approved by the institutional review board (CSMUH no. CS17132). Cancer diagnosis was verified histologically, and assignment of cancer staging was performed based on the tumor, node, metastasis (TNM) staging system of the American Joint Committee on Cancer (AJCC) [28] at the time of diagnosis. Clinicopathological parameters, including liver cirrhosis, vascular invasion, staging and size of the tumor, metastatic status, positivity for HBV surface antigen (HBsAg) and antibody against HCV (anti-HCV), and Child–Pugh grade were collected from the chart reviews. Diagnosis of cirrhosis was made by liver biopsy, ultrasound screening, or blood tests for liver parenchymal damage with endoscopic esophageal or gastric varices. A total of 1195 participants with the same ethnicity who have no self-reported history of cancer of any sites and not diagnosed with HCC were recruited for comparisons. Non-cancer participants with pregnancy and liver transplant recipients were excluded in the control group. In this study, informed written consent was collected from all subjects.

### 2.2. Demographic Information

A survey concerning sex, age, and status of drinking and smoking was collected from all subjects. Consuming more than two alcoholic beverages per day on average is considered habitual drinking. Cigarette smokers are defined as individuals who consumed more than one tobacco-related products/day in the latest season.

### 2.3. Genotyping

DNA samples were isolated by QIAamp DNA blood mini kits (Qiagen, Valencia, CA, USA). Genotyping for three SNPs (rs6983267 for *CCAT2*, rs3843549, and rs13281615 for *CASC8*) was carried out through the TaqMan assay with an ABI StepOne™ Real-Time PCR System (Applied Biosystems, Foster City, CA, USA) and processed with SDS software version 3.0 (Applied Biosystems, Foster City, CA, USA).

### 2.4. Statistical Analysis

Comparisons of demographic parameters between HCC and non-cancer group were conducted by Fisher’s exact test and Mann–Whitney U test. We used a goodness-of-fit v2 test to evaluate the Hardy–Weinberg principle for each genetic marker. The impact of genotypic differences on HCC risk was evaluated using adjusted odds ratios (AORs) with their 95% confidence intervals (CIs) by multiple logistic regression methods after controlling for other covariates, including age, gender, positivity for HBsAg, cigarette smoking, and alcohol drinking. Data were processed using SAS statistical software (Version 9.1, 2005; SAS Institute Inc., Cary, NC, USA). Statistical significance was determined by a *p* value less than 0.05.

## 3. Results

In the present study, we have enrolled 397 HCC patients and 1195 gender-matched normal controls for comparisons. The average age for HCC patients at the disease onset is 63.1 ± 11.5, which is significantly higher than that of normal controls (59.4 ± 7.1) at the beginning of enrollment (Table 1). In addition, we observed that individuals who are positive for HBsAg and anti-HCV, or habitually drinking alcohol had increased susceptibility to HCC. Yet, distribution of participants who smoke habitually did not differ (*p* = 0.845) between two study cohorts.

To assess the effect of genetic variants within the 8q24 region on the occurrence of HCC, three SNPs (rs6983267 for *CCAT2*, rs3843549, and rs13281615 for *CASC8*) were genotyped and analyzed for their relationships with the predisposition to HCC (Table 2). For three SNPs examined, deviation from Hardy–Weinberg equilibrium was obtained in neither case nor control cohort (*p* > 0.05). In addition to odds ratio (OR) with 95% CI, the genetic effect was determined by (AOR), which was calculated by multiple logistic regression models after adjustment for potential confounders in each comparison. Among these loci examined, carrier of rs6983267GG in *CCAT2* were more susceptible to HCC, with the OR and AOR being 1.532 (95% CI, 1.103–2.129; *p* = 0.011) and 1.627 (95% CI, 1.120–2.265; *p* = 0.033), respectively. Yet, genotypic frequencies detected for rs3843549 and rs13281615, individually, did not differ between the controls and cases.

Although no correlation between *CASC8* gene polymorphisms (rs3843549 and rs13281615) and HCC risk was observed, further patient stratification revealed association of these two SNPs with clinical features of liver cancer. Specifically, for patients who are under the age of 65 (Table 3) or male only (Table 4), carriers of at least one minor allele of rs3843549 (heterozygote and homozygote for the minor allele, G) were significantly less prone to develop large-size tumors and had a marginal effect on protection from the development of late-stage tumors. Moreover, patients habitually consuming alcohol and possessing at least one polymorphic allele of rs3843549 were associated with less frequent vascular invasion and lower Child–Pugh scores (Table 5). Similarly, a low Child–Pugh score was often observed in patients who were not smokers and positive for the minor allele of rs13281615 (heterozygous and homozygous for G), indicating a protective effect of rs3843549 and rs13281615 on clinical status of HCC in a dominant inheritance model (Table 6).

## 4. Discussion

Liver tumorigenesis is a complex process attributed to both inherited and environmental factors. Genetic variants within the 8q24 protein-coding gene desert have been associated with many types of malignancies [17,18,23,25,29], yet their impacts on the initiation and progression of HCC remain largely unexplored. In this study, our data show the complexity of lncRNA *CCAT2* and *CASC8* gene variations within the 8q24 region in orchestrating hepatocarcinogenesis.

The oncogenic role of *CCAT2* has been recognized in large part by virtue of an epigenetic mechanism involving its proximity with *MYC* and acting as a sponge through its interaction with some tumor suppressor miRNAs, such as miR-145 [30] and miR-216b [31]. For patients with liver cancer, *CCAT2* has been reported as an oncogene [32], and upregulation of *CCAT2* was detected and associated with poor outcome [33,34]. It has been reported that rs6983267 within *CCAT2* altered the expression level of this lncRNA and conferred the risk of colon cancer [15], implicating rs6983267 as an expression quantitative trait loci (eQTL) in colorectal tissues. Here, we showed a significant association of rs6983267 with elevated susceptibility to liver cancer, although the correlation of *CCAT2* SNPs with HCC risk was not observed in a previous study where the allele(s) of rs6983267 failed to reach Hardy–Weinberg equilibrium [35]. In our investigation, no deviation (*p* > 0.05) from Hardy-Weinberg equilibrium was obtained for rs6983267. We found that carriers of rs6983267GG in *CCAT2*, but not heterozygotes (TG), were more susceptible to HCC after adjustment for possible confounding factors (Table 2). This finding implies that homozygotes for the alternative allele (G) of rs6983267 may have higher expression levels of *CCAT2* transcripts or other oncogenic targets than heterozygotes, implicating rs6983267 as a potential eQTL in hepatic tissues, with a dose effect on influencing the expression levels of its own transcript or target oncogenes.

Unlike *CCAT2* rs6983267 that promotes hepatocarcinogenesis, we observed that variants of rs3843549 and rs13281615 in *CASC8* exhibited a protective effect on the clinical status of HCC. Although both rs3843549 and rs13281615 are located in the intronic region of the *CASC8* gene, recent advances in sequencing technology have uncovered the correlation of numerous intronic variants with human diseases [36,37]. These functional variants commonly reside within the enhancer or silencer and could disturb transcription regulatory motifs and non-coding RNA genes. Consistent with the finding on a role of *CASC8* in suppression of bladder cancer cell proliferation [21], we observed that HCC cases who carry at least one alternative allele of rs3843549 in *CASC8* tend to be protected from developing large-size tumors and against poor functional capacity of the liver (determined by the Child–Pugh grade). These results suggest that variants of two different overlapping lncRNA genes within the 8q24 loci may exhibit both promotive effects on hepatocarcinogenesis as well as protective.

Our data demonstrate an impact of *CCAT2* and *CASC8* gene variations on the occurrence and development of HCC; however, additional efforts are required to address some limitations of the present investigation. First, there are high levels of heterogeneity in HCC-related clinical indices, such as alcoholic and non-alcoholic steatohepatitis within the case group, presumably leading to distinct results concerning the association between *CCAT2* and *CASC8* gene variations and hepatic tumorigenesis. Second, the impacts of external factors on HCC risk could be underestimated because of a lack of cohort stratification attributed to the levels of alcohol consumption. In addition, also lacking is an age-matched control group, since advancing age is a risk for almost all cancer types. Furthermore, the findings detected in this investigation may be constrained to unique ethnic group unless replication experiments are conducted.

## 5. Conclusions

Collectively, data from the present study demonstrated an allelic effect of rs6983267 (*CCAT2*) on conferring the augmented predisposition to HCC. Nevertheless, inverse associations of *CASC8* gene polymorphisms, rs3843549 and rs13281615, with hepatic tumor progression were noted. Our findings unveil the intricate function of overlapping lncRNA genes within the 8q24 region and a dual role of their DNA polymorphisms in developing HCC.

## Figures and Tables

**Table 1:** The distributions of demographical characteristics in 1195 controls and 397 patients with HCC.

Variable	Controls (*n* = 1195)	Patients (*n* = 397)	*p* Value
Age (years)			
Mean ± S.D.	59.4 ± 7.1	63.1 ± 11.3	*p* < 0.001 *
<50	120 (10.0%)	49 (12.3%)	*p* < 0.001 *
50–59	358 (30.0%)	104 (26.2%)	
60–69	700 (58.6%)	125 (31.5%)	
≥70	17 (1.4%)	119 (30.0%)	
Gender			
Male	836 (70%)	276 (69.5%)	
Female	359 (30%)	121 (30.5%)	*p* = 0.870
Cigarette smoking			
No	726 (60.8%)	239 (60.2%)	
Yes	469 (39.2%)	158 (39.8%)	*p* = 0.845
Alcohol drinking			
No	1027 (85.9%)	256 (64.5%)	
Yes	168 (14.1%)	141 (35.5%)	*p* < 0.001 *
HBsAg			
Negative	1049 (87.8%)	227 (57.2%)	
Positive	146 (12.2%)	170 (42.8%)	*p* < 0.001 *
Anti-HCV			
Negative	1142 (95.6%)	219 (55.2%)	
Positive	53 (4.4%)	178 (44.8%)	*p* < 0.001 *
Stage			
I + II		278 (70%)	
III + IV		119 (30%)	
Tumor T status			
T1 + T2		283 (71.3%)	
T3 + T4		114 (28.7%)	
Lymph node status			
N0		385 (97%)	
N1 + N2 + N3		12 (3%)	
Metastasis			
M0		378 (95.2%)	
M1		19 (4.8%)	
Child-Pugh grade			
A		321 (80.9%)	
B or C		76 (19.1%)	
Liver cirrhosis			
Negative		68 (17.1%)	
Positive		329 (82.9%)	

HCC, hepatocellular carcinoma; HBsAg, hepatitis B surface antigen; HCV, hepatitis C virus. Mann–Whitney U test or chi-square test was used between healthy controls and patients with HCC. * *p* value < 0.05 as statistically significant.

**Table 2:** Association of *CCAT2* and *CASC8* gene polymorphisms with HCC.

Variable	Controls (*n* = 1195) *n* (%)	Patients (*n* = 397) *n* (%)	OR (95% CI)	AOR (95% CI) ^a^	Pc
**rs3843549**					
AA	898 (75.1%)	291 (73.3%)	1.000 (reference)	1.000 (reference)	
AG	275 (23%)	96 (24.2%)	1.077 (0.825–1.407)	1.130 (0.835–1.528)	
GG	22 (1.8%)	10 (2.5%)	1.403 (0.657–2.997)	1.417 (0.585–3.429)	
AG + GG	297 (24.9%)	106 (26.7%)	1.101 (0.851–1.426)	1.150 (0.859–1.541)	
**rs6983267**					
TT	416 (34.8%)	118 (29.7%)	1.000 (reference)	1.000 (reference)	
TG	588 (49.2%)	196 (49.4%)	1.175 (0.906–1.525)	1.205 (0.896–1.620)	
GG	191 (16%)	83 (20.9%)	**1.532 (1.103–2.129)**	**1.627 (1.120–2.265)**	**0.033** ^#^
TG + GG	779 (65.2%)	279 (70.3%)	1.263 (0.987–1.615)	1.309 (0.9891.731)	
**rs13281615**					
AA	310 (25.9%)	112 (28.2%)	1.000 (reference)	1.000 (reference)	
AG	600 (50.2%)	195 (49.1%)	0.900 (0.687–1.178)	0.787 (0.578–1.070)	
GG	285 (23.8%)	90 (22.7%)	0.874 (0.634–1.205)	0.735 (0.509–1.062)	
AG + GG	885 (74.1%)	285 (71.8%)	0.891 (0.691–1.149)	0.770 (0.576–1.030)	

HCC, hepatocellular carcinoma. The ORs with their 95% CIs were estimated by logistic regression models. ^a^ Adjusted for the effects of age, sex, HBsAg, cigarette smoking and alcohol drinking. Pc, Corrected *p* value for AOR adjusted by using Bonferroni’s correction ^#^ (*n* = 3). Bold font indicates statistical significance *p* < 0.05.

**Table 3:** Association of clinical status with rs3843549 in *CASC8* among HCC patients under the age of 65.

	Genotypic Frequencies			
Variable	AA (%) (*n* = 155)	AG + GG (%) (*n* = 56)	OR (95% CI)	*p* Value
Clinical Stage				
Stage I/II	103 (66.5%)	45 (80.4%)	1.000 (reference)	
Stage III/IV	52 (33.5%)	11 (19.6%)	0.484 (0.231–1.014)	*p* = 0.054
Tumor size				
≤T2	103 (66.5%)	46 (82.1%)	1.000 (reference)	
>T2	52 (33.5%)	10 (17.9%)	0.431 (0.201–0.921)	*p* = 0.030 *
Lymph node metastasis				
Negative	150 (96.8%)	54 (96.4%)	1.000 (reference)	
Positive	5 (3.2%)	2 (3.6%)	1.111 (0.209–5.897)	*p* = 0.902
Distant metastasis				
Negative	148 (95.5%)	54 (96.4%)	1.000 (reference)	
Positive	7 (4.5%)	2 (3.6%)	0.783 (0.158–3.887)	*p* = 0.765
Vascular invasion				
No	126 (81.3%)	50 (89.3%)	1.000 (reference)	
Yes	29 (18.7%)	6 (10.7%)	0.521 (0.204–1.332)	*p* = 0.174
Child-Pugh grade				
A	120 (77.4%)	47 (83.9%)	1.000 (reference)	
B or C	35 (22.6%)	9 (16.1%)	0.657 (0.293–1.471)	*p* = 0.306
HBsAg				
Negative	75 (48.4%)	28 (50%)	1.000 (reference)	
Positive	80 (51.6%)	28 (50%)	0.938 (0.509–1.728)	*p* = 0.836
Anti-HCV				
Negative	96 (61.9%)	31 (55.4%)	1.000 (reference)	
Positive	59 (38.1%)	25 (44.6%)	1.312 (0.707–2.436)	*p* = 0.389
Liver cirrhosis				
Negative	24 (15.5%)	4 (7.1%)	1.000 (reference)	
Positive	131 (84.5%)	52 (92.9%)	2.382 (0.788–7.199)	*p* = 0.124

> T2: multiple tumors > 5 cm or tumor bearing a major branch of the portal or hepatic vein(s). * *p* value < 0.05.

**Table 4:** Association of clinical status with rs3843549 (*CASC8*) in male HCC patients.

	Genotypic Frequencies			
Variable	AA (%) (*n* = 202)	AG + GG (%) (*n* = 74)	OR (95% CI)	*p* Value
Clinical Stage				
Stage I/II	133 (65.8%)	57 (77%)	1.000 (reference)	
Stage III/IV	69 (34.2%)	17 (23%)	0.575 (0.311–1.063)	*p* = 0.078
Tumor size				
≤T2	132 (65.3%)	59 (79.7%)	1.000 (reference)	
>T2	70 (34.7%)	15 (20.3%)	0.479 (0.254–0.906)	*p* = 0.024 *
Lymph node metastasis				
Negative	194 (96%)	72 (97.3%)	1.000 (reference)	
Positive	8 (4%)	2 (2.7%)	0.674 (0.14–3.247)	*p* = 0.622
Distant metastasis				
Negative	190 (94.1%)	72 (97.3%)	1.000 (reference)	
Positive	12 (5.9%)	2 (2.7%)	0.44 (0.096–2.014)	*p* = 0.290
Vascular invasion				
No	167 (82.7%)	67 (90.5%)	1.000 (reference)	
Yes	35 (17.3%)	7 (9.5%)	0.499 (0.211-1.178)	*p* = 0.112
Child-Pugh grade				
A	162 (80.2%)	63 (85.1%)	1.000 (reference)	
B or C	40 (19.8%)	11 (14.9%)	0.707 (0.341–1.464)	*p* = 0.351
HBsAg				
Negative	102 (50.5%)	42 (56.8%)	1.000 (reference)	
Positive	100 (49.5%)	32 (43.2%)	0.777 (0.455–1.329)	*p* = 0.357
Anti-HCV				
Negative	128 (63.4%)	37 (50%)	1.000 (reference)	
Positive	74 (36.6%)	37 (50%)	1.73 (1.01–2.963)	*p* = 0.046 *
Liver cirrhosis				
Negative	40 (19.8%)	10 (13.5%)	1.000 (reference)	
Positive	162 (80.2%)	64 (86.5%)	1.58 (0.746–3.349)	*p* = 0.232

> T2: multiple tumors > 5 cm or tumor bearing a major branch of the portal or hepatic vein(s). * *p* value < 0.05.

**Table 5:** Association of clinical status with rs3843549 in *CASC8* among HCC patients who habitually consume alcohol.

	Genotypic Frequencies			
Variable	AA (%) (*n* = 96)	AG + GG (%) (*n* = 45)	OR (95% CI)	*p* Value
Clinical Stage				
Stage I/II	66 (68.8%)	36 (80%)	1.000 (reference)	
Stage III/IV	30 (31.3%)	9 (20%)	0.550 (0.235–1.285)	*p* = 0.167
Tumor size				
≤T2	65 (67.7%)	37 (82.2%)	1.000 (reference)	
>T2	31 (32.3%)	8 (17.8%)	0.453 (0.189–1.088)	*p* = 0.077
Lymph node metastasis				
Negative	92 (95.8%)	43 (95.6%)	1.000 (reference)	
Positive	4 (4.2%)	2 (4.4%)	1.070 (0.189–6.068)	*p* = 0.939
Distant metastasis				
Negative	88 (91.7%)	44 (97.8%)	1.000 (reference)	
Positive	8 (8.3%)	1 (2.2%)	0.250 (0.030–2.062)	*p* = 0.198
Vascular invasion				
No	75 (78.1%)	42 (93.3%)	1.000 (reference)	
Yes	21 (21.9%)	3 (6.7%)	0.255 (0.072–0.906)	*p* = 0.035 *
Child-Pugh grade				
A	71 (74%)	40 (88.9%)	1.000 (reference)	
B or C	25 (26%)	5 (11.1%)	0.355 (0.126–0.9998)	*p* = 0.04995 *
HBsAg				
Negative	54 (56.3%)	29 (64.4%)	1.000 (reference)	
Positive	42 (43.8%)	16 (35.6%)	0.709 (0.341–1.474)	*p* = 0.358
Anti-HCV				
Negative	57 (59.4%)	20 (44.4%)	1.000 (reference)	
Positive	39 (40.6%)	25 (55.6%)	1.827 (0.893–3.736)	*p* = 0.099
Liver cirrhosis				
Negative	16 (16.7%)	5 (11.1%)	1.000 (reference)	
Positive	80 (83.3%)	40 (88.9%)	1.600 (0.547–4.681)	*p* = 0.391

> T2: multiple tumors more > 5 cm or tumor bearing a major branch of the portal or hepatic vein(s).* *p* value < 0.05.

**Table 6:** Association of clinical status with rs13281615 in *CASC8* among HCC patients who do not smoke.

	Genotypic Frequencies			
Variable	AA (%) (*n* = 70)	AG+GG (%) (*n* = 169)	OR (95% CI)	*p* Value
Clinical Stage				
Stage I/II	54 (77.1%)	116 (68.6%)	1.000 (reference)	
Stage III/IV	16 (22.9%)	53 (31.4%)	1.542 (0.809–2.941)	*p* = 0.189
Tumor size				
≤T2	55 (78.6%)	120 (71%)	1.000 (reference)	
>T2	15 (21.4%)	49 (29%)	1.497 (0.773–2.898)	*p* = 0.231
Lymph node metastasis				
Negative	68 (97.1%)	164 (97%)	1.000 (reference)	
Positive	2 (2.9%)	5 (3%)	1.037 (0.196–5.474)	*p* = 0.966
Distant metastasis				
Negative	68 (97.1%)	159 (94.1%)	1.000 (reference)	
Positive	2 (2.9%)	10 (5.9%)	2.138 (0.456–10.02)	*p* = 0.335
Vascular invasion				
No	61 (87.1%)	140 (82.8%)	1.000 (reference)	
Yes	9 (12.9%)	29 (17.2%)	1.404 (0.627–3.144)	*p* = 0.409
Child-Pugh grade				
A	51 (72.9%)	143 (84.6%)	1.000 (reference)	
B or C	19 (27.1%)	26 (15.4%)	0.488 (0.249–0.956)	*p* = 0.037 *
HBsAg				
Negative	45 (64.3%)	92 (54.4%)	1.000 (reference)	
Positive	25 (35.7%)	77 (45.6%)	1.507 (0.848–2.677)	*p* = 0.162
Anti-HCV				
Negative	35 (50%)	97 (57.4%)	1.000 (reference)	
Positive	35 (50%)	72 (42.6%)	0.742 (0.424–1.298)	*p* = 0.296
Liver cirrhosis				
Negative	10 (14.3%)	32 (18.9%)	1.000 (reference)	
Positive	60 (85.7%)	137 (81.1%)	0.714 (0.330–1.544)	*p* = 0.392

> T2: multiple tumors > 5 cm or tumor bearing a major branch of the portal or hepatic vein(s). * *p* value < 0.05.
